# Modelling the impact of dietary diversity on child nutrition in Pakistan: a machine learning analysis with Shapley Additive exPlanations and Boruta interpretability

**DOI:** 10.7189/jogh.16.04182

**Published:** 2026-06-12

**Authors:** Muhammad Shahid, Jiayi Song, Muhammad Ali Yahya, Hasan Dincer, Serhat Yuksel, Hafiz Muhammad Naveed, Muhammad Ali

**Affiliations:** 1 College of Management, Shenzhen University, Shenzhen, Guangdong, China; 2 Department of Family Medicine, McGill University, Quebec, Montreal, Canada; 3 Department of Artificial Intelligence, The Islamia University of Bahawalpur, Bahawalpur, Punjab, Pakistan; 4 School of Business, İstanbul Medipol University, Istanbul, Turkey; 5 College of Management Science and Engineering, Shandong University of Finance and Economics, Jinan, Shandong, China; 6 Department of Economics, Al-Madinah International University, Kuala Lumpur, Malaysia

## Abstract

**Background:**

Pakistan faces a serious challenge in malnutrition, as its core nutrition indicators and minimum dietary diversity remain below the recommended level. Here, we investigate the association between the food/dietary diversity index and the nutritional status of children under five years of age and identify influential factors associated with child nutritional status. We hypothesise that poor dietary diversity, *i.e.* the consumption of fewer than five food groups, is a risk factor, while the consumption of five or more groups is a protective factor for child nutrition.

**Methods:**

We used national representative survey data on 4499 children from the 2018 Pakistan Demographic and Health Survey and, through the application of a hybrid machine learning framework for mixed mechanisms, analysed the relationship between the predictive model’s performance and its indicators using machine learning-based logistic regression (ML-LR). We used Shapley Additive exPlanations (SHAP) to assess the model’s characteristics and selected key risk factors through a Boruta algorithm.

**Results:**

We observed that stunting (38.13%), underweight (23.04%), and wasting (8.05%) remain widespread in Pakistan. The ML-LR model identified living in poor areas (Sindh, Balochistan, and federally administered tribes), high birth order, older age, low dietary diversity, recent diarrhoea cases, and maternal unemployment as important risk factors. The SHAP analysis identified the marginal effects of each predictor and confirmed that child age, maternal underweight, and unimproved water were the main risk drivers, while adequate dietary diversity (over five food groups), higher maternal education level and male gender were protective factors. The Boruta algorithm identified low dietary intake, the child’s higher age, and the mother's low nutritional status as the most important determinants among 13 selected factors.

stunting (38.13%), underweight (23.04%), and wasting (8.05%) remain widespread in Pakistan. The ML-LR model identified living in poor areas (Sindh, Balochistan, and federally administered tribes), high birth order, older age, low dietary diversity, recent diarrhoea cases, and maternal unemployment as important risk factors for child nutrition. In this sense, the SHAP analysis identified the marginal effects of each predictor and confirmed that child age, maternal underweight, and unimproved water as the main risk drivers for child nutrition, while adequate dietary diversity (over five food groups), higher maternal education level, and male gender were protective factors. The Boruta algorithm identified low dietary intake, the child’s higher age, and the mother's low nutritional status as the most important determinants among 13 selected factors.

**Conclusions:**

We found that dietary diversity is a key threshold in shaping child nutritional status. Children consuming fewer than five food groups were shown to be at higher risk of malnutrition, while those consuming five or more food groups had improved nutritional outcomes. Public health interventions should prioritise strategies to ensure that this food diversity threshold is met, while also addressing potential socioeconomic constraints. As a secondary finding, we suggest that a combination of ML-LR, SHAP, and Boruta provides a robust, explanatory, and replicatory analytical framework for research in nutrition epidemiology and public health through predicting malnutrition, assessing its characteristics, and identifying its key predictive factors.

Malnutrition remains an urgent global health issue, especially in developing countries, where it accounts for half of all deaths among children under the age of five [1,2]. The World Health Organization (WHO) recommends intake from at least five of eight food groups as a minimum threshold for nutritional adequacy, while emphasising the crucial role of early nutrition and called for exclusive breastfeeding and timely provision of supplementary nutrition as the main references for infant nutrition [3]. These priorities reflect the goal of Sustainable Development Goal 2, which aims to end hunger through improved access to nutrition [4]. Despite global efforts, compliance with recommended dietary standards remains low, and in resource-limited areas, less than a quarter of children aged 6–23 months have access to a fully diversified diet [5]. This problem is compounded by the socioeconomic obstacles many families face in meeting the criteria for diversity [6].

Dietary diversity supports child growth through multiple biological pathways: increased intake of essential micronutrients (iron, zinc, vitamin A) critical for immune function and linear growth; improved protein quality and quantity; and enhanced gut microbiome diversity, reducing enteric dysfunction. Adequate dietary diversity, *i.e.* the consumption five or more varieties (*i.e.* cereals, legumes, dairy products, animal proteins and vitamin-rich agricultural products [7,8], serves as a key indicator of nutritional adequacy and could lead to reductions in child morbidity and mortality. It supports child growth through multiple biological pathways: increased intake of essential micronutrients (iron, zinc, vitamin A) critical for immune function and linear growth; improved protein quality and quantity; and enhanced gut microbiome diversity, reducing enteric dysfunction. Inadequate food diversity, in turn, has been related to a greater risk of stunting, underweight, and wasting among children [9], especially in resource-limited populations that still have limited access to diversified foods [2,10]. Current data show that only 25% of children in developing countries meet the WHO's minimum food diversity requirements [11], despite clear evidence that improving food diversity can significantly enhance their nutritional status, while the lack thereof can lead to poor development, as well as higher susceptibility to anaemia, recurrent infections, and other serious health complications [12].

Recent research indicates that minimum dietary diversity (MDD) is extremely low in Pakistan (14.2%), with the lowest acceptability at 3.6% [13,14]. These deficiencies are among the leading causes of stunting (38.13%), wasting (8.05%) and underweight (23.04%) in children, according to the 2018 Pakistan Demographic Health Survey (PDHS) [15]. Although nutritional diversity, as measured by at least five of the eight major food groups [7,8], is a proven strategy to combat malnutrition, numerous obstacles hinder its adoption in resource-limited contexts [6,9].

Despite the growing recognition of the importance of MDD, there are still major knowledge gaps regarding its impact on the nutritional health of children in Pakistan. Existing research has primarily relied on traditional statistical methods, with little uptake of advanced machine learning technology. For example, studies have yet to utilise hybrid machine learning approaches for estimating malnutrition determinants within the 2018 PDHS [15], which is the broadest health survey data set in Pakistan.

We aimed to address these gaps by proposing two hypotheses: that a poor dietary diversity score (*i.e.* the consumption of fewer than five food groups) significantly increases the odds of child malnutrition, while a greater dietary diversity score (*i.e.* the consumption of more than five food groups) protects against it; and that hybrid machine learning techniques, namely ML-LR in combination with Shapley Additive exPlanations (SHAP) and Boruta algorithms, provide superior interpretability and predictive performance in recognising determinants of malnutrition within complex survey data when compared to conventional regression methods.

We also wanted to examine the following questions: ‘How common are stunting, wasting and underweight in Pakistani children below five years of age?’; ‘Is dietary diversity related to the nutritional status of children, and if so, what is this relationship?’; ‘Which socioeconomic, demographic, and environmental predictors are the most predictive of malnutrition?’; and ‘Can machine learning perform well by combining logistic regression with SHAP and Boruta in terms of identifying malnutrition drivers and explaining their effects?’.

## METHODS

The 2017–2018 PDHS, implemented in cooperation of the Ministry of Services, Organization, and Coordination of Pakistan and the United States Agency for International Development from 22 November 2017 to 30 April 2018, covered the entire population of Pakistan, aiming to provide accurate estimates of health, nutrition, and the overall population size. Its sample is based on the 2017 population and housing census of Pakistan. First, 580 primary sampling units (clusters) were selected in two stages based on the 2017 population and housing census of Pakistan. Then, 28 households are selected from each of the 580 enumeration areas, for a total of 16 240 households. Among these, approximately one-third underwent a nutrition assessment, including 4449 children under five years of age, whose anthropometric measurements were then taken. Using this data, we calculated the weight-for-age (WAZ), weight-for-height (WHZ), and height-for-age (HAZ) data on children aged 0–59 months. This PDHS data allows for precise approximations at the nationwide and regional levels and precisely reflects urban *vs.* rural inhabitants, as it is obtained through multi-stage cluster sampling method.

After excluding cases with biologically implausible Z-scores (<−5 or>+5, per WHO guidelines), those missing anthropometric data, and those with incomplete dietary records, we retained 4098 children in our analytical sample. To improve the reliability of our estimates, we used survey weights (based on the household sample weight recorded in the PDHS, divided by 1 000 000). We also accounted for clustering effects by including primary sampling units in our analysis. We then stratified by geographic region to adjust our models for geographic variation.

### Dependent and independent variables

Using data from the 2017–2018 PDHS [15], we analysed child malnutrition using three growth indicators set by the WHO in 2009 [16]. These include: chronic malnutrition identified through height-for-age deficits (stunting), combined acute/chronic malnutrition reflected in WAZ measures (underweight), and acute malnutrition detected *via* WHZ ratios (wasting). Each indicator uses standard deviation thresholds, where scores falling below −2 SD from the reference median signify clinically significant malnutrition.

To identify overall malnutrition, we implemented the composite index of anthropometric failure (CIAF) which categorises nutritional status into eight mutually exclusive groups based on combinations of stunting, underweight, and wasting [16]. For binary classification, we defined malnutrition as the presence of any anthropometric failure (CIAF categories II–VIII) *vs.* adequate nutrition (CIAF category I, *i.e.* no malnutrition). For statistical modelling purposes, we classified nutritional status as malnutrition (coded as 1) or adequate nutrition (coded as 0).

The key explanatory variable is minimum dietary diversity score. In the PDHS 2018 data [15], the related question is asked and coded in this form: ‘1’ if child receive breast milk, ‘2’ if given potatoes, cassava, or other tuber, ‘3’ if given eggs, ‘4’ if meat (beef, pork, lamb, chicken), ‘5’ if given pumpkin, carrots, squash, ‘6’ if nuts, beans, peas, ‘7’ if vitamin-A rich fruits and vegetables, and ‘8’ if yogurt, other milk products). We defined dietary diversity based on the number of groups consumed in the past 24 hours where, per the WHO infant and young child feeding guidelines [10], the consumption of fewer than five groups indicated inadequate dietary diversity, and the consumption of five or more groups indicated adequate dietary diversity.

We then included demographic, socioeconomic, and environmental variables relevant for child malnutrition, including their gender, early childhood/preschool developmental stage, and their birth order. We also included data on working mothers’ nutritional status, as determined by body mass index (BMI), formal education level, and educational attainment-related parameters.

We examined the household-level predictors separately for urban *vs.* rural areas in all administrative divisions of Pakistan. We measured household wealth as an indicator of economic status. For health, we asked about recent episodes of diarrhoea. For environmental variables, we classified water sources and sanitation facilities as ‘improved’ or ‘unimproved’. Finally, we explored associations of the among dietary diversity index with child malnutrition while controlling for these socioeconomic and environmental variables as potential confounding factors.

### Interpretable machine learning-based analytical framework

#### Machine learning logistic regression

Using logistic regression as the most suitable supervised learning method for predictions with binary labels [17,18], we calculated the risk of malnutrition among children under five years of age in Pakistan. This model obtained a weighted sum of the parameters, which passes through a sigmoid function that results in a value from 0 to 1 and indicate the prediction of the outcome. The first equation represents the traditional logistic regression framework upon which our analysis is based.



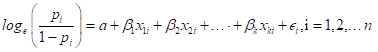



Here, *p_i_* denotes the probability of undernutrition for the *ith* child, 1 − *p_i_* represents the complementary probability of adequate nutrition, *x_ki_* corresponds to the value of the *kth* explanatory variable observed for the *ith* individual participant, while *β_k_* denotes the corresponding parameter estimate that quantifies the association strength of this predictor in the analysis.

Parameter estimation was conducted via maximum likelihood estimation (MLE), which iteratively optimises the log-likelihood function:



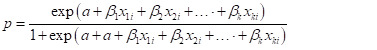





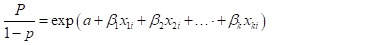



If the odds ratio (OR) of *P*/(1 − *p*) is equal to or greater than 1, the model will classify the child as being malnourished; otherwise, it would classify it as having adequate nutrition. This approach offers several analytical advantages in the context of public health research [17,18]. First, it gives straightforward interpretable estimates of probabilities. Second, the ORs clarify how strongly each factor is associated with malnutrition. Third, this method is well aligned with more conventional nutrition research and allows for ease of comparison between results across studies.

### Data cleaning, model training, tuning, validation, and performance

#### Data cleansing and segmentation

As mentioned above, we excluded cases with biologically implausible Z-scores (<−5 or >+5, per WHO guidelines), resulting in an analytical sample of 4098 children. These were randomly divided into a training set (n = 3073, 75.00%) for model development and a test set (n = 1,025, 25.00%) for final performance assessment. To prevent data leakages, the final test set was separated before any modelling activities.

**Figure 1:**
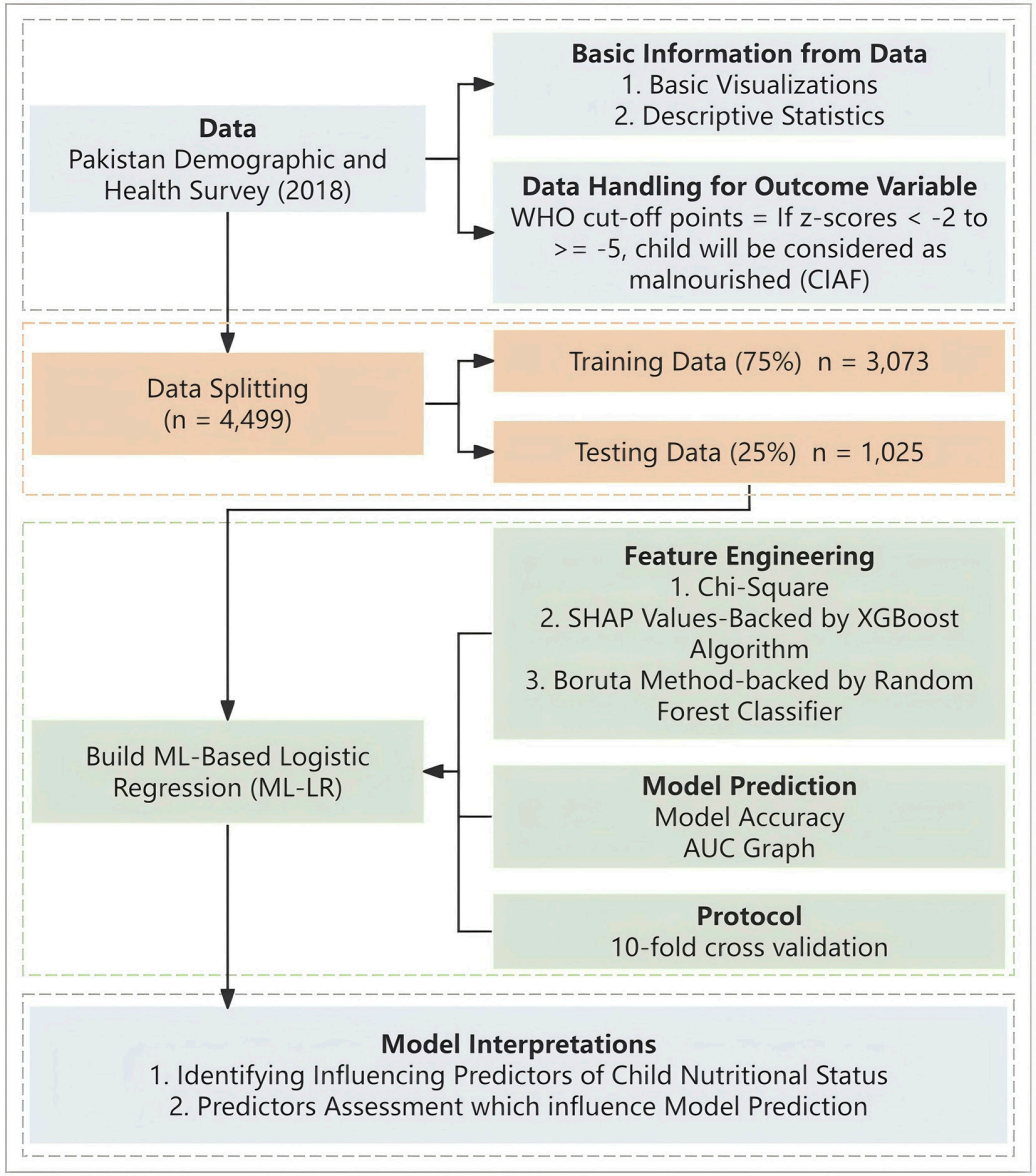
Analytical flowchart.

All analyses were performed using Python, version 3.9.7 (Python Foundation for Statistical Computing, Wilmington, Delaware, USA) and its following libraries: ‘pandas’ (version 1.4.2) for data manipulation, ‘scikit-learn’ (version 1.0.2) for logistic regression and model evaluation, ‘XGBoost’ (version 1.5.0) for SHAP analysis, ‘shap’ (version 0.40.0) for model interpretability, and ‘Boruta’ (version 0.3) for feature selection. The overall analytical workflow of this study is presented in **Figure 1**.

#### Feature scaling

Before model training, we standardised all continuous numerical forecast factors in training groups using ‘Standardskaler’ in the ‘scikit-learn’ library, transforming them to have a mean of zero and a standard deviation of 1. These reduced parameters (mean and SDs) are derived from the training data and are then used in conversion test sets. This step is crucial for logical regression, as it ensures that the model coefficient does not deviate from different ranges of the original properties [19].

We conducted the SHAP analysis using an XGBoost model to capture nonlinear relationships and interactions that logistic regression may miss in phase 1 (*i.e.* the SHAP analysis). During feature selection in phase 2, we used Boruta together with a random forest classifier. Among these three methodologies, the machine learning-based logistic regression (ML-LR) gave interpretable co-efficient and regularised them to reduce the risk of overfitting; SHAP provided insights in terms of marginal effects without being limited to linear assumptions; and the Boruta allowed us to systematically identify true predictors. Using all three methods (*i.e.* ML-LR; SHAP, and Boruta) instead of just one, we were able to get a comprehensive understanding of the drivers behind malnutrition in children.

#### Tuning hyperparameters and training models

We used ordinary least squares and regularised ML-LR models, fine-tuning two of their parameters to improve prediction accuracy: penalty type (L1 or L2; both penalty types were used in our analyses) and regularisation strength (denoted as ‘C’, with multiple of its values examined: 0.001, 0.01, 0.1, 1, 10, and 100). We peprformed 10 rounds of cross-validation and selected the configuration corresponding to the highest receiver operating characteristic (ROC) area under the curve (AUC) average.

#### Evaluating the final model

Once optimal hyperparameter combinations were determined, we retrained the model using all of the training data set (3073 observations). Furthermore, we made predictions on the 1025 cases in the test set, where we computed the ROC curve, accuracy, sensitivity, specificity, and confusion matrix. These metrics represent the model’s performance and validate our approach by ensuring that we used the same data split throughout both analyses (SHAP backed by XGBoost and Boruta algorithm based on random forest).

### Evaluating and selecting predictors using SHAP and Boruta

#### Predictor evaluation using SHAP

We used SHAP alongside an XGBoost model to better understand the predictors that significantly influenced our model [20]. The SHAP’s approach is based on cooperative game theory, a formalism first defined by Lundberg [21], and guarantees that the attributions of influence are both self-consistent and fair.

Unlike other interpretability tools like the Local Interpretable Model-Agnostic Explanations, the SHAP gives a better view of the inner workings of a machine learning model [21]. Because of the mathematics involved in calculating SHAP values, we found that a positive value was associated with an increased risk of malnutrition and that a negative value implied a protective association, indicating adequate nutrition. This dichotomy enabled us to systematically stratify risk factors and protective components in our meta-analysis.

#### Selection of predictors by Boruta

We used Boruta for feature selection, which is a random forest-based extension [22]. Boruta works by producing ‘shadow’ copies of the original variables via random permutation of their values and runs a comparative analysis between each real variable and its perturbed counterpart. The variables that always perform better than their shadow counterparts are left, and those that never meet this condition are discarded. This process ensures that the most pertinent predictors are highlighted, stripping away irrelevant variables, which can cloud model interpretability and damage performance.

Boruta evaluates variable importance by the two metrics, *i.e.* mean decrease accuracy and mean decrease impurity. A variable is considered to be important if its importance score is greater than the maximum score of the shadow features. Although Boruta has a good reputation for detecting relevant predictors, the computational power needed could be high (especially for large data sets) since multiple random forest models must be fit when using this method as part of the feature selection process. Hence, during implementations, a trade-off must be made between methodological rigour and computational expediency [22].

## RESULTS

### Descriptive statistics

**Figure 2:**
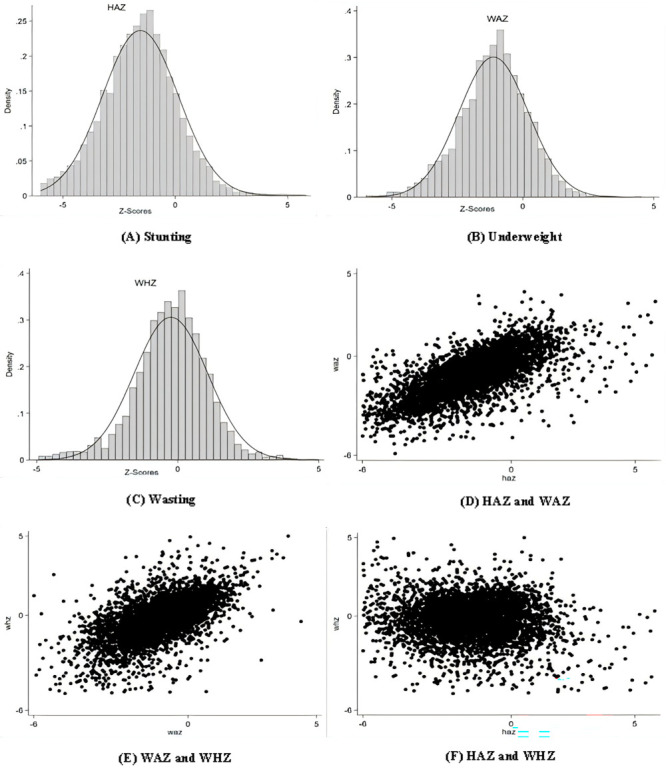
Distribution of z-scores and correlation among child anthropometric indicators.

We found a large sample of children who lie in both the moderate and severe categories of stunting, wasting, and underweight (Z-scores between −2 and −5) (**Figure 2**, Panels A–C). We also observed significantly high prevalence rates for growth retardation (HAZ<−2 SD) and low body mass relative to age (WAZ<−2 SD), and a low prevalence of acute undernutrition (WHZ<−2 SD). We otherwise found a positive association between HAZ and WAZ, and between WAZ and WHZ, as well as a weak correltaion between HAZ and WHZ (**Figure 2**, Panels D–F). In detail, age-adjusted weight and height metrics were significantly correlated (r = 0.42; *P* < 0.001), and there were strong associations between WHZ and WAZ (r = 0.68; *P* < 0.001), as well as between WAZ and height-for-age (r = 0.61; *P* < 0.001). Malnutrition prevalence was markedly higher among children with lower dietary diversity (Figure S1 in the **Online Supplementary Document**), while the malnutrition rate was significantly lower among children meeting the dietary diversity threshold (*P* < 0.01).

The prevalence of malnutrition was 22.49% among boys and 21.67% among girls (**Table 1**). It was the highest in children aged 25–36 months (10.43%) and 37–48 months (10.33%). Children with birth order above two years had higher malnutrition rates (18.1%). Among children with recent diarrhoea, malnutrition prevalence was 35.04%. Geographically, malnutrition prevalence was higher in rural areas (26.23%) than in urban areas (17.91%). Provincial prevalence varied from 10.16% in Sindh, 7.45% in Balochistan (7.45%), 7.03% in Khyber Pakhtunkhwa, to 7.25% in Punjab. Furthermore, the prevalence of malnutrition was 38.58% among children with unemployed mothers, 27.99% among mothers with no formal education, and 39.62% among children whose mothers were underweight (BMI < 18.5 kg/m^2^). By wealth quintile, malnutrition prevalence was 13.44% in the richer quintile and 12.37% in the poorer quintile. We found a 34.92% prevalence of malnutrition among the group which lacked improved water supply and 31.36% among those who lacked improved sanitation. Among children consuming fewer than five food groups, 78.8% were malnourished.

**Table 1:** Sociodemographic and nutritional characteristics of study participants (n = 4098)

	n (%)*	*P*-value†
**Child characteristics**		
Sex		0.67
*Female*	888 (21.67)	
*Male*	922 (22.48)	
Age in months		<0.001
*0–12*	284 (6.95)	
*13–24*	325 (7.93)	
*25–36*	423 (10.32)	
*37–48*	427 (10.44)	
*49–60*	349 (8.51)	
Birth order in years		<0.001
*≤2*	766 (18.71)	
*3–4*	548 (13.38)	
*5–7*	379 (9.24)	
*>7*	115 (2.82)	
Recent diarrhoea		<0.001
*Yes*	1436 (35.05)	
*No*	372 (9.11)	
Dietary diversity of food groups		<0.001
*<5*	1425 (78.80)	
*≥5*	383 (21.20)	
**Household characteristics**		
Residence		<0.001
*Rural*	1075 (26.24)	
*Urban*	733 (17.91)	
Region		<0.001
*Sindh*	416 (10.16)	
*Punjab*	297 (7.25)	
*Balochistan*	305 (7.45)	
*Khyber Pakhtunkhwa*	288 (7.03)	
*FATA*	189 (4.62)	
*Azad Jammu & Kashmir*	142 (3.47)	
*Gilgit Baltistan*	108 (2.64)	
*Islamabad Capital*	63 (1.54)	
Drinking water		<0.001
*Unimproved*	1431 (34.93)	
*Improved*	377 (9.22)	
Sanitation		<0.001
*Unimproved*	1285 (31.37)	
*Improved*	523 (12.79)	
Wealth quintile		<0.001
*Poorest*	550 (13.44)	
*Poorer*	507 (12.37)	
*Middle*	324 (7.92)	
*Richer*	254 (6.21)	
*Richest*	173 (4.21)	
**Maternal characteristics**		
Nutritional status (BMI)		<0.001
*Underweight (<18.5 kg/m^2^)*	181 (39.62)	
*Normal (≥18.5 kg/m^2^)*	1615 (4.44)	
Education		<0.001
*No formal education*	1146 (27.99)	
*Primary*	234 (5.71)	
*Secondary*	290 (7.08)	
*Higher*	138 (3.37)	
Employment		<0.001
*Not employed*	1579 (38.58)	
*Employed*	229 (5.59)	

*Weighted percentages account for complex survey design.

†*P*-values were derived from Rao-Scott χ^2^ tests.

### Output of ML-LR

Older children had significantly higher malnutrition risk compared to the reference group (0–12 months), with the highest odds among children aged 25–36 months (OR = 2.38; 95% confidence interval (CI) = 1.93–2.88, *P* < 0.001). Higher birth order was associated with increased risk (OR = 1.22 for birth order 3–4; OR = 1.33 for birth order 5–7; *P *< 0.001). Recent diarrhoea increased malnutrition risk (OR = 1.27; 95% CI = 1.07–1.48, *P *= 0.001). Significant regional disparities were observed: Sindh (OR = 2.06; 95% CI = 1.66–2.55), Balochistan (OR = 2.53; 95% CI = 1.96–3.27), and Khyber Pakhtunkhwa (OR = 1.28; 95% CI = 1.04–1.65) showed higher risk compared to Punjab (*P* < 0.001 for all). Urban residence was protective (OR = 0.78; 95% CI = 0.68–0.92, *P* = 0.001). Normal maternal BMI (≥18.5 kg/m^2^) was protective (OR = 0.79; 95% CI = 0.63–0.99, *P* = 0.001). Higher maternal education (OR = 0.52; 95% CI = 0.38–0.67, *P* < 0.001) and maternal unemployment (OR = 1.29; 95% CI = 1.05–1.59, *P* = 0.001) were associated with malnutrition risk. Improved water supply (OR = 0.78; 95% CI = 0.67–0.96, *P* = 0.001) and improved sanitation (OR = 0.58; 95% CI = 0.53–0.71, *P* < 0.001) were protective, as was higher wealth (richest quintile OR = 0.31; 95% CI = 0.24–0.42, *P* < 0.001). Dietary diversity below five food groups was associated with increased malnutrition risk (OR = 1.18; 95% CI = 1.06–1.55, *P* < 0.001) (**Table 2**).

**Table 2:** Machine learning-based logistic regression results for child undernutrition (CIAF)*

	OR (95% CI)	*P*-value
**Children's biological sexual orientation**		<0.001
Female	ref	
Male	1.045 (0.93–1.18)	0.48
**Children's age group in months**		0.01
0–12	ref	
13**–**24	1.59 (1.28–1.98)	0.01
20**–**36	2.38 (1.93–2.88)	0.01
37**–**48	2.33 (1.88–2.84)	0.01
48**–**60	1.94 (1.57–2.37)	0.01
**Birth order classification in years**		0.001
≤2	ref	
3**–**4	1.22 (1.05–1.41)	0.03
5**–**7	1.33 (1.11–1.58)	0.001
>7	1.35 (1.02–1.83)	0.002
**Residence location**	ref	<0.001
Rural		
Urban location	0.78 (0.68–0.92)	0.04
**Region/province**		0.004
Punjab	ref	
The Sindh region/province	2.06 (1.66–2.55)	0.89
The Khyber Pakhtunkhwa region/province	1.28 (1.04–1.65)	0.88
The Balochistan region/province	2.53 (1.96–3.27)	0.46
The FATA region/province	1.54 (1.17–2.03)	0.004
The Gilgit Baltistan region/province	1.03 (0.77–1.39)	0.03
The Islamabad-Capital region	0.98 (0.68–1.38)	0.88
The Azad Jammu and Kashmir region/province	0.91 (0.79–1.19)	0.007
**Maternal health, referred to as BMI**		0.001
<18.5 kg/m^2^	ref	
≥18.5 kg/m^2^, indicating normal status	0.79 (0.63–0.99)	0.03
**Recent occurrence of diarrhoea in a child**		<0.001
No	ref	
Yes	1.27 (1.07–1.48)	0.12
**The drinking water classification**		0.001
Unsafe	ref	
Safe	0.78 (0.67–0.96)	0.001
**Sanitation facilities type**		<0.001
Unimproved	ref	
Strengthened or improved	0.58 (0.53–0.71)	0.001
**Maternal education**		<0.001
Illiterate	ref	
Primary education	0.86 (0.69–1.05)	<0.001
Secondary education	0.76 (0.62–0.92)	0.005
Higher education	0.52 (0.38–0.67)	0.001
**Employment status of women**		<0.001
Employed	ref	
Unemployed	1.29 (1.046–1.59)	0.001
**Household wealth index/status**		<0.001
Lowest wealth group	ref	
Lower-class category	0.92 (0.54–1.66)	0.48
Middle-class category	0.53 (0.43–0.68)	0.001
Richer-class category	0.41 (0.33–0.54)	0.001
Richest-class category	0.31 (0.24–0.42)	0.001
**Dietary diversity index**		<0.001
Child having >5 food groups		
Child receiving <5 food groups	1.18 (1.06–1.55)	0.001

AUC – area under the curve, BMI – body mass index, CI – confidence interval, FATA – federally administered tribal areas, OR – odds ratio, ROC – receiver operating characteristic

*Machine learning-based logistic regression model performance, evaluated on 2052 observations, showed an accuracy of 86%, sensitivity of 79.06%, specificity of 77.38%, precision of 0.789, an F1-score of 0.865, and an AUC-ROC of 0.90.

The ML-LR model achieved strong discriminatory performance with an AUC-ROC of 0.90 and an overall classification accuracy of 86% (Figure S2, Panel A in the **Online Supplementary Document**). While the model correctly classified 1919 children as not malnourished (true negatives) and 207 children as malnourished (true positives), it misclassified 119 children as malnourished when they were not (false positives) and 144 children as not malnourished when they were malnourished (false negatives) (Figure S2, Panel B in the **Online Supplementary Document**).

**Figure 3:**
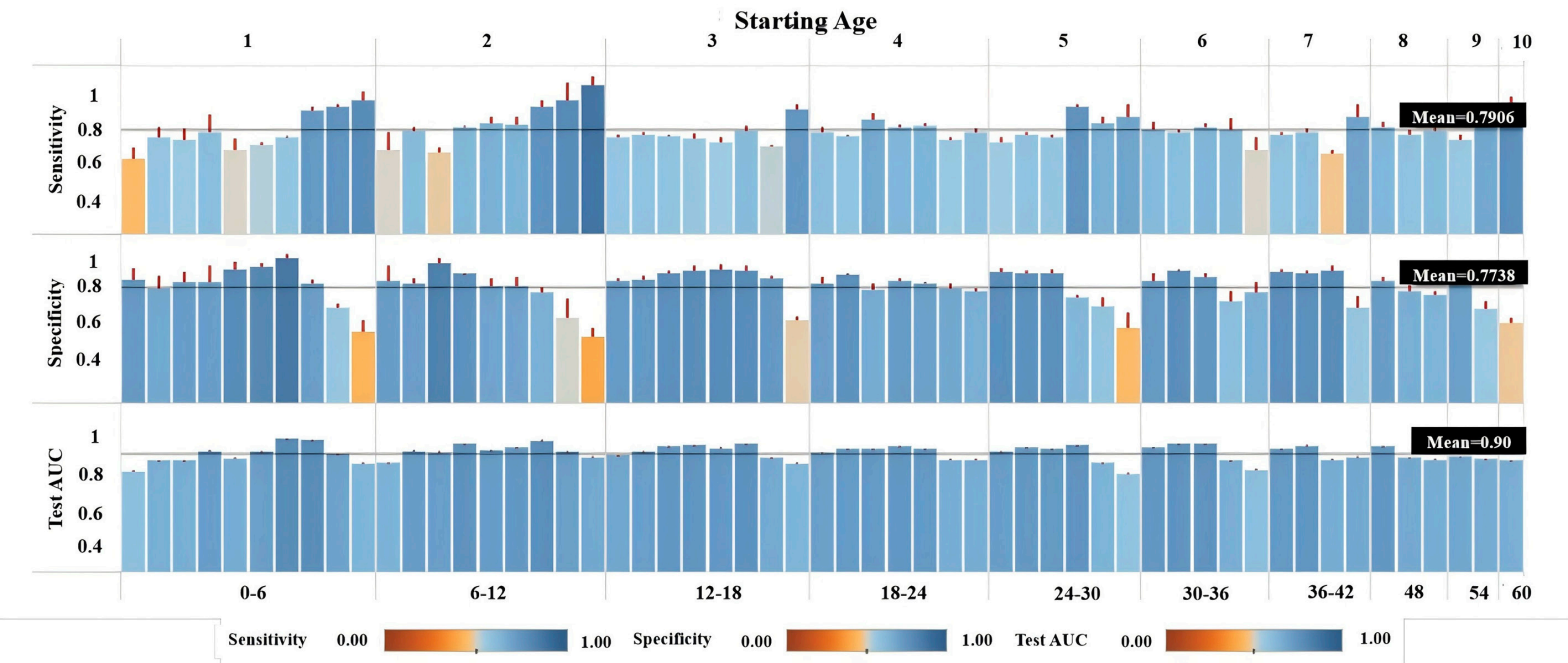
Machine learning-logistic model performance across child age ranges and data length.

**Figure 3** dismantles the assumption of model stationarity in child malnutrition prediction. When the LR model was disaggregated by age, performance was not uniform across strata. The average test AUC-ROC across age cohorts reached 0.90, yet this aggregate mask clinically meaningful variation: mean sensitivity (0.7906) and specificity (0.7738) followed opposing age-specific trajectories. Critically, these age-stratified estimates bear little resemblance to the pooled ML-LR model's global ROC-AUC or confusion matrix, confirming that whole-population LR metrics overestimate generalizability and conceal age-specific predictive blind spots. In essence, age acts as a non-linear effect modifier – the LR model’s predictive accuracy for malnutrition is not transferable across developmental windows without substantial recalibration.

### SHAP and Boruta methodology interpretation for selection and evaluation of prediction factors

**Figure 4:**
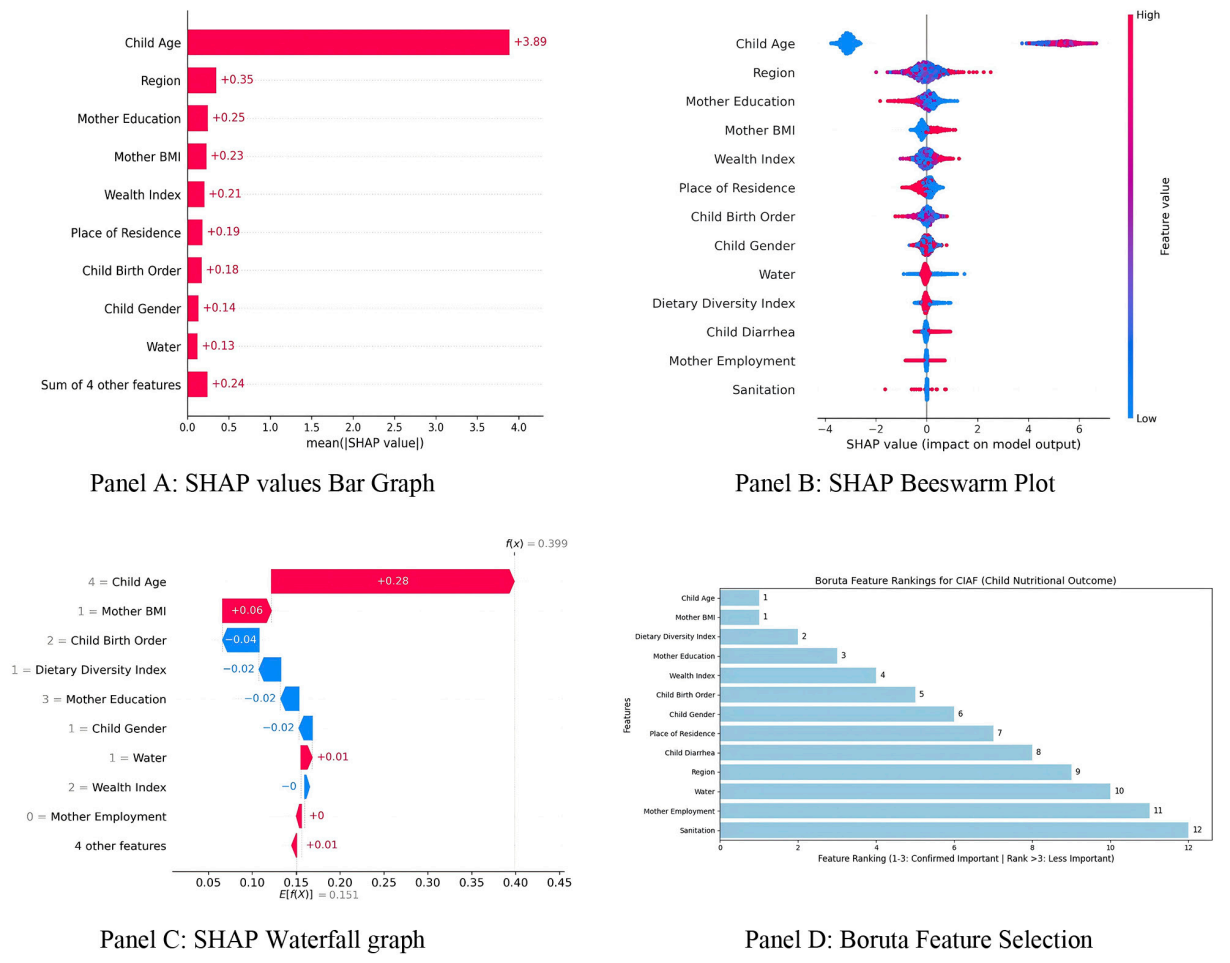
Predictors assessment and risk factors identification through SHAP and Boruta approach.

This study uses visual regression analysis using SHAP and Boruta analysis for model interpretability and risk factors identification (**Figure 4**).

The SHAP analysis identified the following predictors as increasing malnutrition risk: older child age, maternal underweight (BMI <18.5 kg/m^2^), maternal unemployment, and unimproved water sources (**Figure 4**, Panels A–C). Higher birth order, lack of dietary diversity (fewer than five food groups), and male gender were also associated with increased risk. In contrast, higher maternal education was associated with reduced risk. The Boruta algorithm confirmed dietary diversity (fewer than five food groups), child age, maternal underweight (BMI <18.5 kg/m^2^), and low maternal education as important predictors of child nutritional status in Pakistan (**Figure 4**, Panel D).

## DISCUSSION

Our study highlights the associations of various food consumption with childhood undernutrition in Pakistan. Unlike previous research, which primarily examined dietary diversity as a continuous predictor in econometric models, our analysis explicitly identifies a threshold effect: consuming fewer than five food groups emerges a risk factor for malnutrition, while consuming five or more food groups is shown to be a protective factor. Furthermore, our SHAP analysis quantifies the marginal contribution of each predictor to the model's predictions, providing deeper insight than traditional association measures. This fits with the existing literature from Pakistan. For example, one study found that consuming five food groups as nutritional benchmark to be especially relevant for the country, since its traditional diet greatly depends on stable grains for energy, but has an insufficient intake of protein foods, vegetables, and fruits [23]. Food insecurity, economic constraints, and low nutrition-related knowledge play major determinants of the unhealthy dietary pattern of communities, especially in rural areas [24–26]. The association of lower number food groups consumed with higher odds of malnutrition can be explained further with low access to different foods during the year faced by many Pakistani families. This is exacerbated for bedridden patients, those who are undergoing treatment or agricultural landholders that have multiple seasons of rising food scarcity and price fluctuations [27,28]. Nevertheless, the protection associated with five or more food groups was similar to that reported for other studies demonstrating that even modest improvement in dietary diversity (the consumption of dairy products and legumes) or certain specific foods can lead to higher micronutrient intake and better growth [27,28]. These findings are reinforced by the National Nutrition Survey and the PDHS, which consistently report that less than one-third of children meet the WHO Minimum Dietary Diversity standards [29]. Similarly, studies in India, Indonesia, and China indicate that adequate dietary diversity is always associated with reduced rates of malnutrition [30–32]. Similar patterns were observed in several African countries, including Ethiopia, Ghana, Kenya, the Democratic Republic of the Congo, South Africa, and Tanzania, reinforcing the global importance of food diversity in the fight against stunting and malnutrition [33–39].

Besides nutritional factors, we identified several socioeconomic and health determinants of child malnutrition through advanced machine learning techniques (ML-LR, SHAP, and Boruta): recent diarrhoea, older child age, maternal undernutrition, low maternal education, household poverty, and poor water access. These findings are consistent with previous studies in Pakistan on the interactions between maternal health and economic status and child nutrition [40–48]. The control factors we used in our study are interconnected and highlight the need for integrated approaches that take into account diet diversity, as well as the underlying socioeconomic and environmental determinants of child nutrition [19,49–51].

We further consolidated machine learning-based logistic regression with SHAP and Boruta feature selection. The resulting model was not only predictive but also easily interpretable. This framework allows policymakers to identify which predictors (*e.g.* low dietary diversity, maternal underweight, unimproved water) have the greatest marginal impact on malnutrition risk, enabling targeted allocation of limited resources to the most vulnerable populations in resource-limited settings.

### Practical uses for the findings of this study

Our study provides policymakers and public health workers with an integrated methodology for analysing the problem of child malnutrition in Pakistan. The results suggest that minimum diversity (*i.e.* children's access to the equivalent of five or more food items) is essential to prevent and treat malnutrition, but must be complemented by interventions targeting socio-economic determinants. Specifically: nutrition projects should prioritise increasing food diversity through community education, subsidised food baskets, and behavioural change campaigns that emphasise the importance of providing children with adequate dietary diversity. Social protection policies should address maternal health, education, and employment issues, as they have a significant impact on the nutritional status of children. Conditional cash transfers or maternal nutrition support projects can mitigate these risks [50].

Simultaneously, the SHAP and Boruta-enhanced ML frameworks could be replicated further in Pakistan and similar regions for identifying high-risk groups and (consequently) optimising resource allocation. The national monitoring system could use these predictive models to monitor malnutrition trends and assess the dynamic effectiveness of the programme.

### Contributions of this research

We offer three substantive contributions to the fields of nutrition research and policy, especially for child health. First, methodological precision is improved, through the introduction of an innovative machine learning framework combining SHAP (Shapley Additive Explanations), Boruta and logistic regression algorithms. This study provides a way for achieving better predictive accuracy of the risk assessment and forecasts, with an interpretable outcome as a side benefit. This approach manages the challenging scenario of unbalanced dietary data presenting in child- health determinants. Second, empirical evidence: our analysis, based on national representative data, provides strong evidence that food diversity (less than 5 *vs.* ≥5 dietary items intake) is an adjustable threshold that affects the consequences of child malnutrition in Pakistan. We measure the influence of this on the nourishing status of children through its interaction with socio-economic factors, such as the maternal quality index, education and the Wash condition. Third, the policy perspective: research goes beyond links or traditional analytical methods to find possible points of intervention. Research suggests that improved nutritional diversity should be combined with nutrition, education and water for pregnant women and postpartum women in order to effectively reduce malnutrition. These investments have contributed to enhanced academic understanding and practical interventions to address child malnutrition in resource-constrained Settings. Nutritional researchers could improve agricultural analysis based on the methodology of collecting this study in command to advance the presentation of the research.

### Limitations and guidance for future research

Although this study provides useful insights into the causes of child undernourishment in Pakistan, many of these determinants essential be taken into consideration when understanding the outcomes. First, the review and observation of demographic and health survey data have hindered the causal relationship between specific risk factors and nutritional outcomes. Second, food diversity data based on maternal self-responses are subject to potential reporting biases that may affect accuracy. Third, our analysis was limited by the available variables in the PDHS, while possible determinants such as detailed household food safety, nutritional input, and seasonal variations in food supply were unavailable.

Furthermore, the main systemic constraint remains the lack of external certification: the reported performance metrics, such as the AUC-ROC of 0.90, are fragmented from the internal training tests in the 2017–2018 PDHS data set. Therefore, although the model has demonstrated strong predictive power on these specific data, it may not spread to new independent population groups or different periods. These measurements can be thus be considered optimistic. The effectiveness of the true deployment model in the real world has yet to be determined through external verification. Second, the cross-sectional design of the PDHS prevents causal inference. Our findings should be interpreted as associations rather than causal relationships. Longitudinal studies following children over time are needed to establish causality. Third, our analytical framework used three different machine learning models: ML-LR for prediction, XGBoost for SHAP analysis, and random forest for Boruta feature selection. While each model serves a specific purpose, the lack of a unified framework means that feature selection, prediction, and interpretability are not derived from a single consistent process. Future research could develop an integrated approach where feature selection and prediction originate from the same model to ensure methodological coherence. Studies with proactive approaches need to be conducted to better define the causal relationship between food diversity and child's development. First, in order to make predictions more accurate, the ML framework should provide context with data that is more exact on key indicators of nutrition such as ingredient composition, serving size and metric labels. Most importantly, the PDHS predictive model requires validation of external data sets (such as data from health surveys in different regions) in order to assess their generalisability. Finally, intervention studies are needed to assess the actual effectiveness of these policies and plans in field environments, based on automated learning models.

## CONCLUSIONS

This study presents a robust analysis of the PDHS 2018 data and shows that inadequate dietary diversity (consumption of fewer than five food groups) is a significant risk factor for child malnutrition, while adequate dietary diversity (consumption of five or more food groups) is a protective factor. We also found that low maternal BMI, maternal illiteracy, poor access to clean drinking water, and higher birth order were associated with increased odds of malnutrition. In contrast, higher maternal education and children consuming five or more food groups were associated with improved nutritional outcomes.

These findings highlight two essential priorities. First, public health interventions should ensure that children achieve adequate dietary diversity by consuming at least five food groups daily. This can be accomplished through targeted nutrition education for caregivers, food supplementation programs, and conditional cash transfers linked to dietary diversity goals. Second, interventions must simultaneously address interrelated structural barriers, including maternal health (*e.g.* nutritional support for underweight mothers), maternal education (*e.g.* girls’ schooling and adult literacy programs), and environmental conditions (*e.g.* improving access to clean water and sanitation facilities). The machine learning framework we developed, which integrates ML-LR with SHAP and Boruta, can be utilised for effective risk prediction and policy intervention planning, including integration into national nutrition monitoring systems in Pakistan.

## Additional material


Online Supplementary Document
