# Supplementary material for: Modelling the impact of dietary diversity on child nutrition in Pakistan: a machine learning analysis with Shapley Additive exPlanations and Boruta interpretability
Source: J Glob Health. 2026 Jun 12;16:04182. doi: 10.7189/jogh.16.04182 (PMC13261326; doi:10.7189/jogh.16.04182)
Supplement: Online Supplementary Document [file jogh-16-04182-s001.pdf]

Supplement to: Shahid M, Song J, Yahya MA, Dincer H, Yuksel S, Naveed HM, Ali M. Modelling the impact of dietary diversity on child nutrition in Pakistan: a machine learning analysis with Shapley Additive exPlanations and Boruta interpretability. J Glob Health. 2026;16:04182.

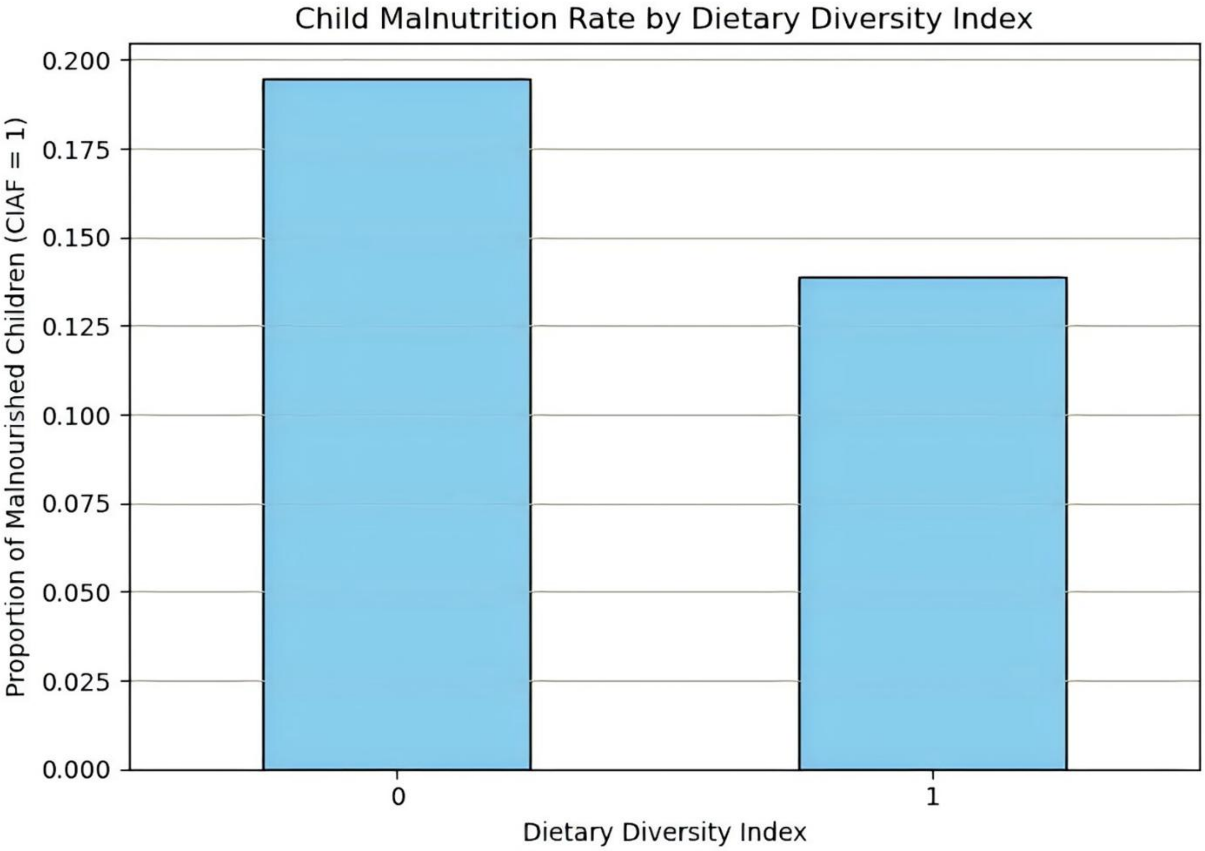

Supplementary Figure S1. Interaction of dietary diversity index and child malnutrition

In Figure S1, the Y-axis depicts the proportion of malnourished children, and X-axis represents the bars by dietary diversity where “0” presents in-adequate dietary diversity unable to meet minimum food requirements, while “1” represents the adequate dietary meeting minimum standards (equal to and greater than 5 food groups).

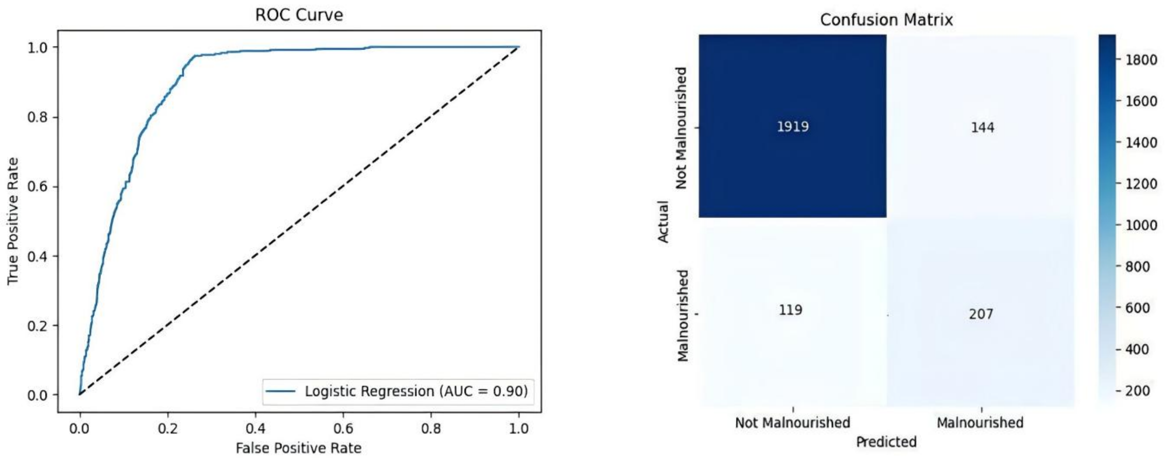

Panel (A): ML-LR ROC curve

Panel (B): ML-LR Confusion Matrix

Supplementary Figure S2. ML-LR ROC curve and confusion matrix

In Figure S2, Panel (A) represents the AUC-ROC curve of machine learning-based logistic regression model (ROC value=0.90, accuracy=86%). While Panel (B) of Figure S2 shows the Confusion matrix of ML-LR classification, where model correctly classified 1,919 children as not malnourished and it misclassified 119 children as malnourished when they were not.
